# A prospective observational study on trajectories and prognostic factors of mid back pain

**DOI:** 10.1186/s12891-020-03534-5

**Published:** 2020-08-17

**Authors:** Christina Knecht, Sonja Hartnack, Beate Sick, Fabienne Riner, Petra Schweinhardt, Brigitte Wirth

**Affiliations:** 1 Integrative Spinal Research, Department of Chiropractic Medicine, University Hospital Balgrist, Forchstr. 340, 8008 Zurich, Switzerland; 2 Section of Epidemiology, Vetsuisse Faculty, University of Zurich, Winterthurerstr. 270, 8057 Zurich, Switzerland; 3 University of Zurich, Epidemiology, Biostatistics & Prevention Institute (EBPI), Hirschengraben 84, 8001 Zurich, Switzerland

**Keywords:** Chiropractic, Cluster, Improvement, Mid back, Outcome, Pain, Risk factor, Spine, Thoracic, Trajectory

## Abstract

**Background:**

Although mid back pain (MBP) is a common condition that causes significant disability, it has received little attention in research and knowledge about trajectories and prognosis of MBP is limited. The purpose of this study was to identify trajectories of MBP and baseline risk factors for an unfavorable outcome in MBP patients undergoing chiropractic treatment.

**Methods:**

This prospective-observational study analyzes outcome data of 90 adult MBP patients (mean age = 37.0 ± 14.6 years; 49 females) during one year (at baseline, after 1 week, 1 month, 3, 6 and 12 months) after start of chiropractic treatment. Patients completed an 11-point (0 to 10) numeric pain rating scale (NRS) at baseline and one week, one month, three, six and twelve months after treatment start and the Patient’s Global Impression of Change (PGIC) questionnaire at all time points except baseline. To determine trajectories, clustering with the package kml (software R), a variant of k-means clustering adapted for longitudinal data, was performed using the NRS-data. The identified NRS-clusters and PGIC data after three months were tested for association with baseline variables using univariable logistic regression analyses, conditional inference trees and random forest plots.

**Results:**

Two distinct NRS-clusters indicating a favourable (rapid improvement within one month from moderate pain to persistent minor pain or recovery after one year, 80% of patients) and an unfavourable trajectory (persistent moderate to severe pain, 20% of patients) were identified. Chronic (> 3 months) pain duration at baseline significantly predicted that a patient was less likely to follow a favourable trajectory [OR = 0.16, 95% CI = 0.05–0.50, *p* = 0.002] and to report subjective improvement after twelve months [OR = 0.19, 95% CI = 0.07–0.51, *p* = 0.001], which was confirmed by the conditional inference tree and the random forest analyses.

**Conclusions:**

This prospective exploratory study identified two distinct MBP trajectories, representing a favourable and an unfavourable outcome over the course of one year after chiropractic treatment. Pain chronicity was the factor that influenced outcome measures using NRS or PGIC.

## Background

Mid back pain (MBP) or thoracic spine pain, described as pain between the 1st and 12th thoracic vertebrae and the corresponding posterior aspect of the trunk [1] is a common condition in the general adult population (12-month prevalence rate 15–35% in the general adult population; median in most occupational groups around 30% [2, 3]). Compared to low back pain (LBP) (12-month prevalence rates: 1–83%; median 12-month prevalence rate: 37% [4]) and neck pain (NP) (12-month prevalence rates: 17–75%; mean 12-months prevalence rate: 37% [5]), MBP might be slightly less common in the adult population, although it can be equally disabling [3] and result in the same consequences [6, 7]. Nevertheless MBP has received considerably less attention than LBP and NP in research [2] and the thoracic spine has been called the ‘Cinderella region’ or step child of spinal research [8, 9].

A recent systematic review by Johansson and colleagues identified a knowledge gap with respect to MBP recovery trajectories and prognostic factors [10]. Because pain characteristics of the three spinal regions are similar [6, 7], suggesting that spinal pain might be a general disorder, the authors hypothesized that MBP trajectories might be comparable to those of NP and LBP [10]. Regarding prognostic factors, a systematic review on MBP identified only two prospective studies [3] and both focused on MBP in adolescence [11, 12]. In adults, a more recent study reported poor patient expectations for recovery to be a prognostic factor for delayed recovery from traumatic MBP [13], while female gender and other concurrent musculoskeletal symptoms were cross-sectionally associated with MBP [3].

To help closing the identified knowledge gaps in MBP research, the aim of this prospective observational study was to identify trajectories and risk factors for an unfavorable outcome in MBP patients undergoing chiropractic treatment.

## Methods

### Participants and study design

The present study analyzes data collected between June 2014 and December 2019 of an ongoing study tracking patients of a chiropractic teaching clinic affiliated with a mainly orthopedic university hospital in Switzerland. Patients over 18 years without any contra-indications to chiropractic manipulative treatment (e.g. tumors, infections, inflammatory spondylarthropathies, acute fractures and severe osteoporosis) completed an 11-point (0 to 10) numeric rating scale (NRS) for present pain intensity before the first chiropractic treatment (baseline) and after one week, one month and after three, six and twelve months. In addition, they completed the Patient’s Global Impression of Change (PGIC), a seven point Likert-scale assessing the patient’s rating of overall improvement with the extremes “much worse” and “much better” [14] at all time points apart from baseline. Lastly, data on pain duration, previous MBP episodes, presence of concurrent LBP or NP, pain onset (traumatic or non-traumatic), smoking habits and rib involvement (based on the chiropractor’s diagnosis) was assessed. After written informed consent was obtained, the questionnaires were administered to the patients by the treating chiropractor immediately before the first treatment. Patients chose whether they preferred to answer the follow-up questionnaires via email or phone. If phone contact was preferred, a trained research assistant who did not know the patient conducted telephone interviews at each time point, irrespective of whether the patient was still undergoing chiropractic treatment or not. If online contact was chosen, survey invitations were sent to the participants using the software REDCap (version 8.3.2), a secure web-based application designed to support data capture for research studies [15]. This study was approved by the Ethics review board of the Canton of Zurich (EK-16/2009; update PB_2017–00402).

### Data analysis and statistics

Patients for whom NRS data were available from at least five out of the six time points were included. To handle missing data, the random forest package (MissForest [16]), a non-parametric imputation method for mixed-type data, was used. This method uses a random forest trained on the observed data to predict the missing values without the need for a test set or cross validation. In the present study, 4.6% of all values were missing with a maximum of 12 missing values (=13.3%) in the variable ‘PGIC after 1 week’.

Repeated measures ANOVA was conducted to test for the significance of NRS changes between baseline and one year after treatment start. For the PGIC, the two highest categories (“much better” and “better”) were defined as clinically relevant improvement [17, 18]. The McNemar test was used to test whether the percentage of patients who reported improvement significantly changed over time. Descriptive statistical analyses were conducted using the non-imputed data. All inferential analyses used the imputed data set.

Clustering with the package KmL (statistical software R 3.4.2; Boston, MA), a variant of k-means clustering specifically designed for longitudinal data, was performed to determine trajectories [19, 20]. The algorithm reaches the optimal cluster number by alternating an ‘expectation phase’, where the center of each cluster is determined, and a ‘maximization phase’ that assigns each observation to its nearest cluster. This alternation is repeated until no further changes in the clusters occur. To determine the optimal number of clusters, five criteria were used to strengthen the reliability of the result (Calinski & Harabatz, Ray & Turi, Davies & Bouldin). These criteria assess the quality of partition by combining the between-cluster covariance and the within-cluster covariance [19, 20].

The obtained clusters were subsequently tested for association with baseline factors using univariable logistic regression analysis with cluster membership as the dependent variable. The independent variables were age, gender, pain duration (acute/subacute versus chronic; acute and subacute patients were summarized into one category because medium (1 month) and long-term (≥ 3 months) outcome was shown to be comparable for these groups in NP [21]), previous episodes (no previous episode versus ≥1 previous episodes), other painful areas (no concurrent versus concurrent LBP or NP or headache), whether the treating chiropractor diagnosed rib involvement (costo-vertebral syndrome yes/no), traumatic onset (yes/no), and smoking status (yes/no). To test the effects of these baseline factors on the patients’ perception of outcome (PGIC) after twelve months, univariable binary logistic regression models with the dependent variable PGIC (dichotomized; ‘improved’ = better or much better, rest = ‘not improved’) were run. The univariable logistic regression approach was chosen because the statistical power was too low to include all these potential risk factors in the same multivariable regression model and the approach of using univariable analyses as an initial step to select covariates for further consideration in a multivariable model was shown to be invalid as this might lead to biased estimates [22]. Instead the multivariable model was investigated using conditional inference trees [23–25] and random forest plots [26]. Tree-based methods are an alternative to logistic regression to reveal risk factors [27] and are increasingly used in clinical problems [28]. To test for multicollinearity, pairwise associations were tested with Fisher’s exact tests.

Descriptive statistics, repeated measures ANOVA and logistic regression analyses were performed using SPSS 24.0 (IBM Corp., Chicago, IL). Data imputation, determination of trajectories, the conditional inference trees and random forest plots were performed using the statistical software R (statistical software R 3.4.2; Boston, MA [29]). For all analyses, a *p*-value of < 0.05 was considered statistically significant.

## Results

Ninety patients (mean age = 37.0 ± 14.6 years) of whom 49 (54.4%) were female were included in the analysis (84 patients were excluded due to fewer than five data sets). Of these, 15 patients chose to be contacted via phone and 75 patients answered the follow-up questionnaires using the provided electronic link. For the majority of patients (*N* = 57/64.8%), the current MBP episode was the first, while 11 patients (12.5%) had suffered from one to three previous episodes and 20 patients (22.7%) from more than three episodes (2 missing values). Fourty-eight (53.9%) patients were acute (pain duration ≤30 days), 8 (9.0%) subacute patients (pain duration 31–90 days) and 33 (37.1%) chronic patients (pain duration > 90 days) (1 missing value). Thirty-four patients (37.8%) reported concurrent LBP, NP or LBP and NP. Ten patients (11.4%) reported MBP of traumatic origin (2 missing values), and rib involvement was reported by the treating chiropractor in 36 patients (40.0%). Of the 52 patients without rib involvement, 25 (27.8%) were diagnosed with axial pain and 29 (32.2%) were diagnosed with somatic referred pain. Twenty-four patients (27.0%) were smokers (1 missing value). These patients were comparable to those who were excluded from the study because they did not provide data of at least five of the six time points and did not statistically differ in any of the variables mentioned above.

**Fig. 1:**
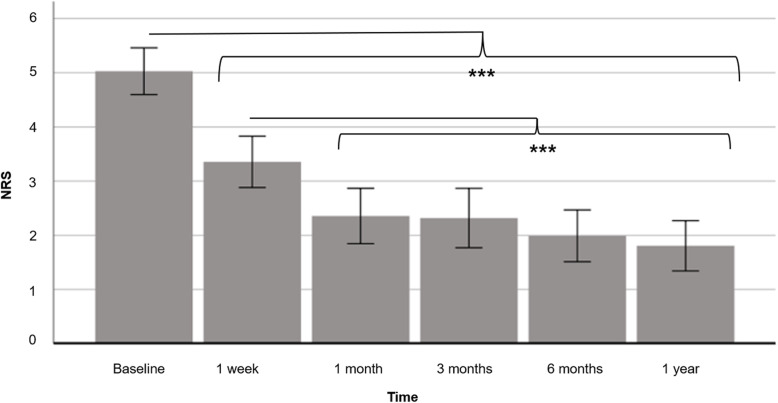
Pain intensity at baseline and one week, one month, three months, six months and one year after treatment start. The values in the numeric rating scale (NRS) significantly improved within the first year after start of chiropractic treatment from 5.03 (± SD 2.06) points at baseline to 3.36 (± SD 2.26) after one week, to 2.36 (± SD 2.44) after one month, to 2.32 (± SD 2.61) after three months, to 1.99 (± SD 2.28) after six months and to 1.81 (± SD 2.21) points after one year. Pain reduction was significant from baseline to all time points (p’s < 0.001) and from one week to all time points (p’s < 0.001). The error bars represent ±1 standard deviation. NRS=Numeric rating scale; ***p < 0.001

NRS values significantly improved within the first twelve months after start of chiropractic treatment from 5.03 (± SD 2.06) points at baseline to 1.81 (± SD 2.21) points [F_(192.93, 3.56)_ = 54.16, *p* < 0.001]. Post-hoc tests showed significant pain reduction from baseline to all time points (p’s < 0.001) and from one week to all time points (p’s < 0.001). Afterwards, no significant pain reduction occurred up to one year after treatment start (Fig. 1).

The percentage of patients reporting improvement in the PGIC significantly increased from *N* = 38/48.7% after one week (12 missing values) to *N* = 58/70.7% after one month (8 missing values) (*p* < 0.001), where it stabilized [improvement after three months *N* = 60/69.8%, 4 missing values; after six and twelve months N = 60/72.3%, 7 missing values]. Using the imputed data set, the results were comparable (improvement = 45.6% after 1 week, 70.0% after 1 month, 71.1% after 3, 6 and 12 months).

### Trajectories

**Fig. 2:**
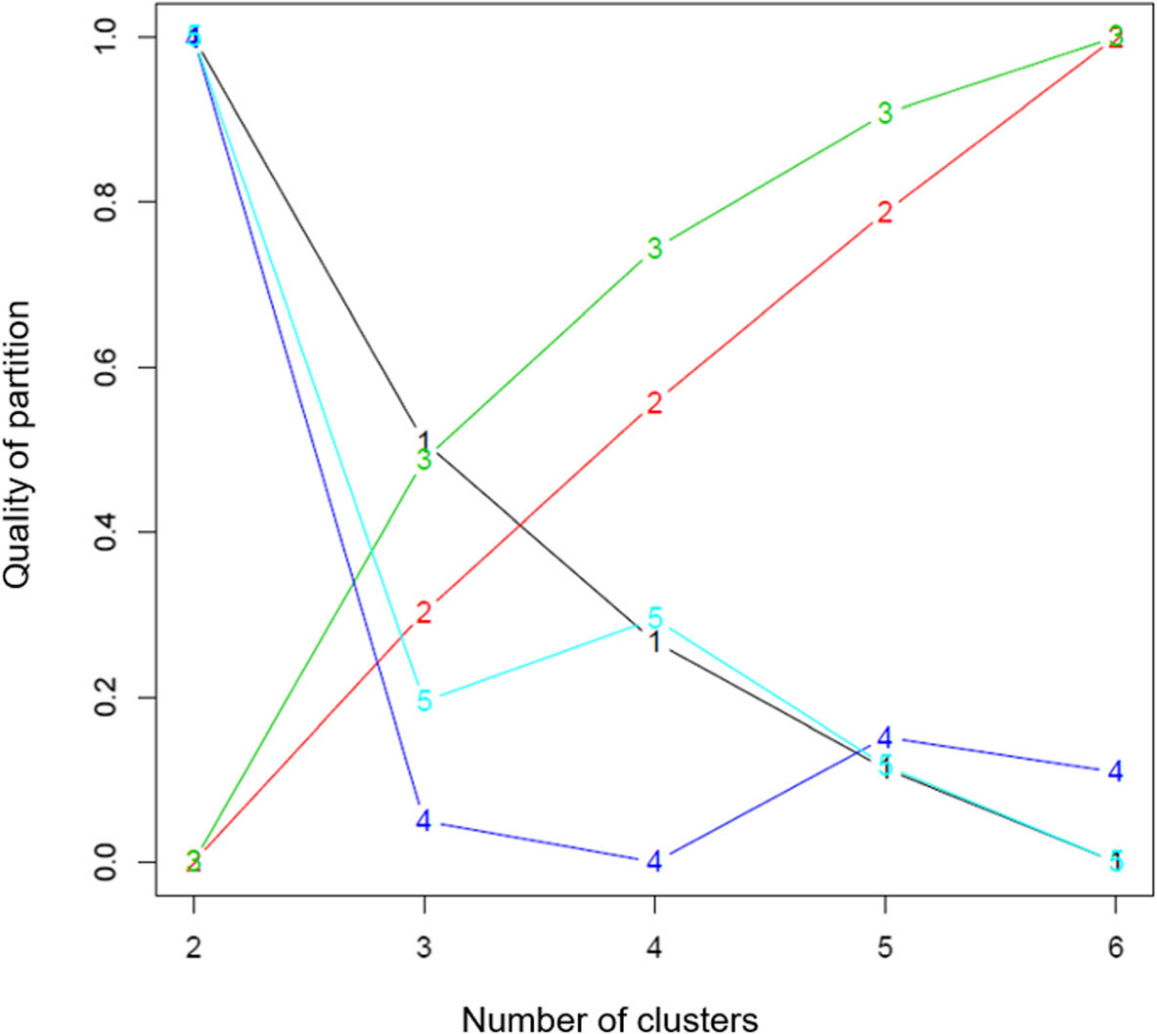
Partition quality of cluster analysis. The x-axis shows the number of clusters, the y-axis the quality of the partition, which represents a combination of the within- and the between-cluster covariance. A high value indicates good partition quality. A two-cluster solution was favored by three criteria (1 = Calinski Harabatz 1; 4 = Ray Turi; 5 = Davies Bouldin), a six-cluster solution by two criteria (2 = Calinski Harabatz 2; 3 = Calinski Harabatz 3)

The five criteria indicated highest partition quality for a two- and six-cluster solution: three criteria were in favor of two clusters and two criteria in favor of six clusters (Fig. 2).

**Fig. 3:**
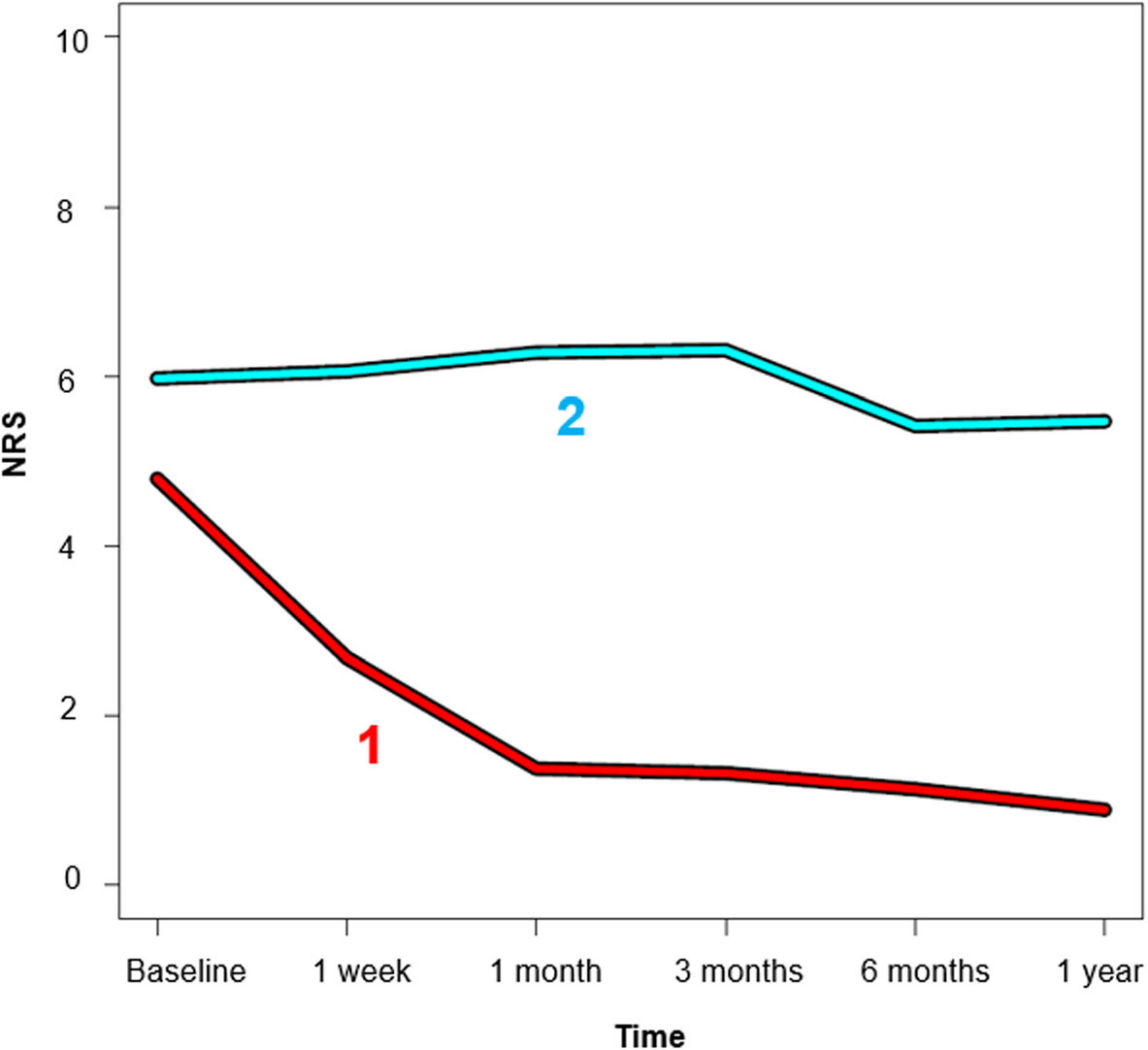
Two-cluster solution. Trajectory 1 (red) represents the ‘improvers’ (rapid recovery from moderate pain and persistent minor pain), trajectory 2 (blue) represents the ‘non-improvers’ (persistent moderate pain)

Using the terminology by Kongsted and colleagues for LBP trajectories [30], the trajectory 1 (‘improvers’) in the two-cluster solution describes rapid improvement within one month from moderate pain to persistent minor pain or recovery [30] after one year. The trajectory 2 (‘non-improvers’) describes persistent moderate to severe pain [30]. The majority of patients (*N* = 72/80%) followed trajectory 1 (*N* = 51/70.8% were acute/subacute), and 18 patients (20%) followed trajectory 2 (N = 5/27.8% were acute/subacute). Thus, 91.1% from the acute/subacute patients and 61.8% from the chronic patients followed trajectory 1 (Fig. 3). Of note, these numbers can slightly vary when choosing different seeds in the model due to the stochastic nature of the analysis.

**Fig. 4:**
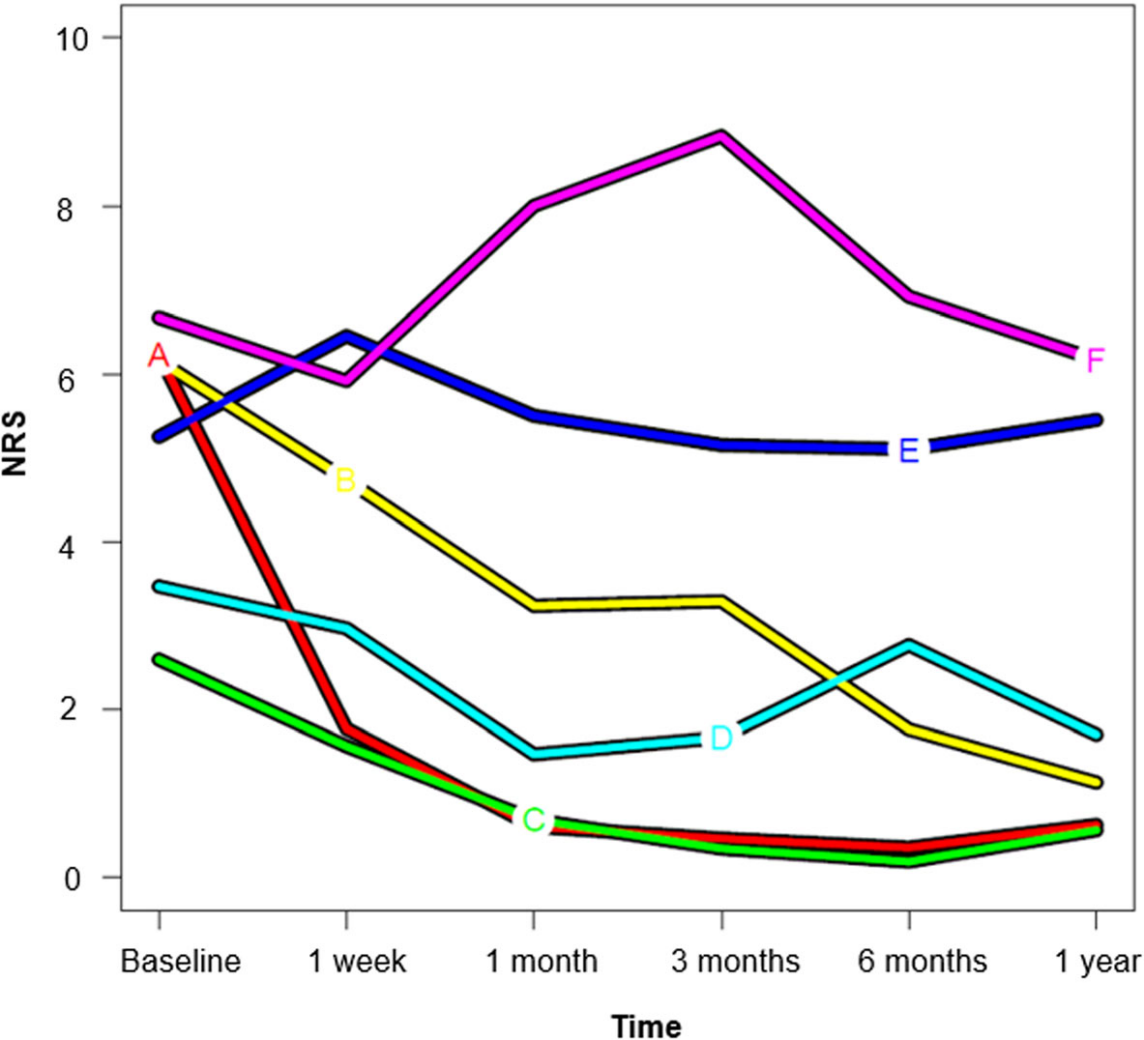
Six-cluster solution. The trajectories describe rapidly improving pain, starting either from mild (Cluster C, green), moderate (Cluster D, light blue) or from severe pain (Cluster A, red; Cluster B, yellow) describe rapidly improving pain (‘marked decrease in pain intensity within one month’ [30]) followed by recovery/minor pain (Cluster A and C) or mild pain (Cluster B and D) [30] after one year with one cluster (Cluster D) showing some slight pain exacerbation at three months

In the six-cluster solution selected by two criteria with the same high partition quality as the two-cluster solution, four clusters, starting either from mild (Cluster C), moderate (Cluster D) or from severe pain (Cluster A and B) describe rapidly improving pain (‘marked decrease in pain intensity within one month’ [30]) followed by recovery/minor pain (Cluster A and C) or mild pain (Cluster B and D) [30] after one year with one cluster (Cluster D) showing some slight pain exacerbation at six months (Fig. 4). Of the acute patients, 91–95% followed one of these trajectories, while 59–65% of the chronic patients showed one of these pain patterns. On the higher end of the NRS-scale, two clusters (Cluster E and F) describe persistent moderate to severe pain [30]) after 12 months, either after prior gradual improvement or some temporary exacerbation. These pain patterns were followed by 5–9% of the acute and 35–41% of the chronic patients.

### Predictive factors

Traumatic onset (Nagelkerke R^2^ = 0.09) and chronic pain (Nagelkerke R^2^ = 0.18) were the factors that predicted the unfavorable trajectory (trajectory 2) in the univariable logistic regression models based on NRS data (Table 1). The factors age, gender, previous pain episodes, concurrent pain areas, rib involvement, and smoking status were not found to influence the trajectory.

**Table 1:** Predictors for trajectory. Results of univariable binary logistic models

Trajectory 1 (‘improvers’, 1) versus trajectory 2 (‘non-improvers’, 0)	OR (95% CI)	***p***-value
**Age** (years)	1.00 (0.97–1.04)	0.836
**Gender** male (0), female (1)	0.53 (0.18–1.56)	0.249
**Pain duration** acute/subacute ≤3 months (0), chronic > 3 months (1)	**0.16 (0.05–0.50)**	**0.002**
**Previous episodes** no previous episode (0), ≥ 1 previous episodes (1)	1.56 (0.50–4.86)	0.443
**Other areas of complaint** none or other complaints than LBP or neck pain (0), LBP and/or neck pain (1)	0.60 (0.21–1.70)	0.336
**Traumatic onset** no (0), yes (1)	**0.19 (0.05–0.77)**	**0.019**
**Rib involvement** no (0), yes (1)	1.19 (0.41–3.42)	0.749
**Smoker** no (0), yes (1)	0.93 (0.29–2.96)	0.905

*OR* Odds Ratio, *CI* Confidence Interval, *LBP* Low Back Pain. Significant results are marked in bold.

In the conditional inference tree analysis using the same factors as the univariable models as independent variables, chronic pain was the only significant predictor for becoming a ‘non-improver’ (*p* = 0.006), which was confirmed as being the most important factor (highest mean decrease accuracy) in the random forest analysis (estimate of error rate 20.0%). Similarly, pain duration longer than 3 months predicted no improvement in the PGIC 12 months after treatment start in the univariable logistic regression models (Nagelkerke R^2^ = 0.17) (Table 2), and this was confirmed in the conditional inference tree analysis (*p* = 0.002) and in the random forest analysis (estimate of error rate 34.44%). The Fisher’s exact tests for testing pairwise associations found only one significant association, namely between the factors ‘other complaints’ and ‘rib involvement’ (*p* = 0.009) indicating that multicollinearity was a minor problem in the model.

**Table 2:** Predictors for Patient’s Global Impression of Change (PGIC) three months after treatment start. Results of univariable binary logistic models

PGIC after 3 months not improved (0) vs. improved (1)	OR (95% CI)	***p***-value
**Age** (years)	1.01 (0.97–1.04)	0.747
**Gender** male (0), female (1)	0.83 (0.33–2.09)	0.693
**Pain duration** acute/subacute ≤3 months (0), chronic > 3 months (1)	**0.19 (0.07–0.51)**	**0.001**
**Previous episodes** no previous episode (0), ≥ 1 previous episodes (1)	2.28 (0.81–6.45)	0.120
**Other areas of complaint** none or other complaints than LBP or neck pain (0), LBP and/or neck pain (1)	0.87 (0.35–2.21)	0.776
**Traumatic onset** no (0), yes (1)	0.57 (0.15–2.21)	0.415
**Rib involvement** no (0), yes (1)	1.24 (0.49–3.16)	0.645
**Smoker** no (0), yes (1)	0.75 (0.27–2.05)	0.575

*OR* Odds Ratio, *CI* Confidence Interval, *LBP* Low Back Pain. Significant results are marked in bold.

## Discussion

In this population of mostly acute and subacute MBP patients undergoing chiropractic treatment, two trajectories emerged as the optimal solution of the cluster analysis, namely the ‘improvers’ (rapid recovery within one month and full recovery or persistent mild pain, 62%) and the ‘non-improvers’ (persistent moderate pain, 38%). The second best partition was a six-cluster solution revealing a more fine grained representation of trajectories. Pain longer than three months before treatment start significantly predicted that a patient followed the ‘non-improver’ trajectory in the two-cluster solution and reported non-improvement in the PGIC after three months.

### Trajectories

Because of the lack of information in the literature regarding MBP trajectories and prognostic factors [10], the trajectories observed in this study cannot be compared to other MBP studies. However, it has been hypothesized that MBP trajectories might be similar to those of LBP as pain characteristics of the three spinal regions, including NP, are comparable, suggesting that spinal pain is the general underlying disorder [6, 7].

In *acute* LBP patients, Downie and colleagues [31] and Kongsted and colleagues [32] found a majority of patients recovering (70% [31] and 60% [32], respectively), either rapidly or gradually, and a minority of patients with persistent severe pain (5% [31] and 3% [32], respectively). Similarly, a smaller study in 108 patients with a first episode of acute LBP reported recovery in 67% and persistent severe pain in 7% of patients [33]. These findings are compatible with the two-cluster solution (9% of acute MBP patients with persistent severe pain) in the present study. In *chronic* LBP patients, Tamcan and colleagues depicted the course of untreated LBP in the general population and did not observe any improvement (defined as ‘marked decrease in pain intensity’ [30]) [34]. Macedo and colleagues analyzed trajectories over one year in 155 chronic LBP patients of all arms of an RCT on the effects of motor control exercises versus a graded activity intervention using cluster analysis [35]. They identified three clusters, which were gradual improvement (‘marked decrease in pain intensity occurring gradually over more than one month’ [30]) with persistent mild pain in 47%, persistent moderate pain in 25% and persistent high pain in 15% of the patients. These clusters compare well with the clusters A-D (persistent minor or mild pain) and the clusters E and F (persistent moderate or high pain) in the present study. In summary, the MBP trajectories observed here are similar to those described for LBP. Acute spinal pain mostly follows a recovery trajectory with a minority of patients developing persistent or fluctuating pain. In contrast, chronic spinal pain recovers in a minority of patients and persists on a mild, moderate or severe pain level in many instances.

### Factors predictive for poor outcome

Pain duration longer than three months before treatment start was associated with poor outcome in the present study reflected in the patients following an unfavorable trajectory based on NRS data and in the patients reporting no overall improvement in the PGIC, although this association was relatively low, with pain duration explaining 18% (NRS) and 17% (PGIC) of the variance in outcome (R^2^ = 0.18 and 0.17). In accordance, MBP of longer duration (> 30 days) was associated with a higher incidence rate for sickness absence than acute MBP [10]. Overall, the reported improvement was comparable to that of LBP and NP [18, 36] and the finding of higher percentages of self-reported overall improvement in acute compared to chronic MBP supports previous findings in LBP and NP [18, 36]. Altogether, these findings confirm that longer pain duration is a risk factor for poor outcome of musculoskeletal pain as shown in a systematic review [37].

### Limitations

Because the present study design did not include a control group, these data do not allow for drawing any conclusions about the effectiveness of chiropractic treatment. Instead, the data might depict the natural history of MPB. Furthermore, the relatively small number of MPB patients, their heterogeneity with respect to symptom duration and the fact that the MBP patients were recruited from a teaching clinic at a university hospital might limit the generalizability of the present study. Lastly, several factors possibly influencing the course and outcome of MBP, such as other concurrent therapies, medication, pre-existing comorbidities and physical activity were not included in the analysis. With regard to imaging it should be noted that approximately 90% of back problems are non-specific without any pathoanatomical cause that would be identifiable using imaging [38]. Therefore, guidelines discourage from routine imaging in these patients [39].

## Conclusion

This study is a first step towards identifying trajectories in MBP, which were shown to be comparable to those of LBP. Pain duration longer than three months emerged as main risk factor for poor outcome. These findings support the notion that LBP and MBP belong to the same general disorder, which needs being confirmed in larger studies in the future.

## Data Availability

The datasets used are available from the corresponding author upon request.

## Abbreviations

LBP: Low back pain
MBP: Mid back pain
NP: Neck pain
NRS: Numeric pain rating scale
PGIC: Patient’s Global Impression of Change
SD: Standard deviation
